# Supplementary material for: Cognitive training effects are shaped more by individual brain dynamics than age—evidence from younger and older women
Source: GeroScience. 2025 Dec 11;48(5):7095–113. doi: 10.1007/s11357-025-02039-0 (PMC13601467; doi:10.1007/s11357-025-02039-0)
Supplement: Supplementary file 1 [file 11357_2025_2039_MOESM1_ESM.docx]

**GeroScience**

**Supporting Information for**

Cognitive training effects are shaped more by individual brain dynamics than age – Evidence from younger and older women

Zsófia Anna Gaál, Petia Kojouharova, Boglárka Nagy, Gwen van der Wijk, István Czigler, Andrea B. Protzner

Corresponding author: Andrea B. Protzner

Email: protzner@ucalgary.ca

**Table S1.** Summary of the statistical results of the all-group analysis by frequency band. The *Frequency* column indicates the frequency band. The *Compared effects and means* columns show the two compared effects and their averaged similarity, and indicate which effect was the *baseline* for the comparison and which effect was *investigated* for uniquely contributing to the similarity. The *Statistical results* columns show the results of the statistical comparison. Each row shows one dependent *t*-test comparison. The values of *p* are uncorrected, * shows whether the *p* value remained significant after the FDR (False Discovery Rate) correction, and the **bold text** indicates whether the effect was significantly different from and larger than its baseline.

| **Frequency** | **Compared effects and means** | | | | **Statistical results** | | | | | |
| --- | --- | --- | --- | --- | --- | --- | --- | --- | --- | --- |
|  | **First effect (baseline)** | **Mean** | **Second effect (investigated)** | **Mean** | ***t*-test (*df* = 78)** | ***p* (uncorrected)** | ***SD*** | **95% CI (lower)** | **95% CI (upper)** | ***Cohen's d*** |
| **delta (1-4 Hz)** | **Common** | **0.366** | **Session** | **0.368** | **-2.462** | **0.015*** | **0.006** | **-0.003** | **0.000** | **0.277** |
|  | **Common** | **0.366** | **Task** | **0.382** | **-11.844** | **<.001*** | **0.012** | **-0.019** | **-0.013** | **1.333** |
|  | **Common** | **0.366** | **Age** | **0.384** | **-3.836** | **<.001*** | **0.041** | **-0.027** | **-0.008** | **0.432** |
|  | **Common** | **0.366** | **Training** | **0.374** | **-2.634** | **0.008*** | **0.027** | **-0.014** | **-0.002** | **0.296** |
|  | Training | 0.374 | Training × Session | 0.376 | -1.677 | 0.08 | 0.009 | -0.004 | 0.000 | 0.189 |
|  | **Age** | **0.384** | **Training × Age** | **0.396** | **-2.909** | **0.002*** | **0.036** | **-0.020** | **-0.004** | **0.327** |
|  | **Task** | **0.382** | **Training × Task** | **0.391** | **-2.730** | **0.006*** | **0.028** | **-0.015** | **-0.002** | **0.307** |
|  | **Age** | **0.384** | **Session × Age** | **0.389** | **-4.105** | **<.001*** | **0.011** | **-0.008** | **-0.003** | **0.462** |
|  | Task | 0.382 | Session × Task | 0.383 | -0.804 | 0.437 | 0.009 | -0.003 | 0.001 | 0.090 |
|  | **Age** | **0.384** | **Task × Age** | **0.404** | **-12.664** | **<.001*** | **0.014** | **-0.023** | **-0.017** | **1.425** |
|  | **Training × Age** | **0.396** | **Training × Age × Session** | **0.402** | **-2.914** | **0.002*** | **0.019** | **-0.010** | **-0.002** | **0.328** |
|  | Training × Task | 0.391 | Training × Session × Task | 0.389 | 0.996 | 0.317 | 0.013 | -0.001 | 0.004 | -0.112 |
|  | **Task × Age** | **0.404** | **Training × Age × Task** | **0.417** | **-2.883** | **0.007*** | **0.039** | **-0.021** | **-0.004** | **0.324** |
|  | Task × Age | 0.404 | Session × Age × Task | 0.407 | -1.449 | 0.163 | 0.017 | -0.006 | 0.001 | 0.163 |
|  | Training × Age × Task | 0.417 | Training × Session × Age × Task | 0.416 | 0.404 | 0.693 | 0.027 | -0.005 | 0.007 | -0.045 |
|  | **Training × Age × Task** | **0.417** | **Individual** | **0.695** | **-14.766** | **<.001*** | **0.167** | **-0.316** | **-0.241** | **1.661** |
|  | **Individual** | **0.695** | **Individual × Task** | **0.878** | **-8.689** | **<.001*** | **0.188** | **-0.226** | **-0.141** | **0.978** |
|  | Individual | 0.695 | Individual × Session | 0.705 | -0.939 | 0.354 | 0.096 | -0.031 | 0.011 | 0.106 |
|  |  |  |  |  |  |  |  |  |  |  |
|  |  |  |  |  |  |  |  |  |  |  |
|  |  |  |  |  |  |  |  |  |  |  |
|  |  |  |  |  |  |  |  |  |  |  |
|  |  |  |  |  |  |  |  |  |  |  |
| **Frequency** | **Compared effects and means** | | | | **Statistical results** | | | | | |
|  | **First effect (baseline)** | **Mean** | **Second effect (investigated)** | **Mean** | ***t*-test (*df* = 78)** | ***p* (uncorrected)** | ***SD*** | **95% CI (lower)** | **95% CI (upper)** | ***Cohen's d*** |
| **theta (4-7 Hz)** | Common | 0.332 | Session | 0.332 | -0.687 | 0.466 | 0.002 | -0.001 | 0.000 | 0.077 |
|  | **Common** | **0.332** | **Task** | **0.342** | **-10.555** | **<.001*** | **0.009** | **-0.012** | **-0.008** | **1.188** |
|  | **Common** | **0.332** | **Age** | **0.360** | **-6.850** | **<.001*** | **0.037** | **-0.037** | **-0.020** | **0.771** |
|  | **Common** | **0.332** | **Training** | **0.335** | **-4.126** | **<.001*** | **0.008** | **-0.005** | **-0.002** | **0.464** |
|  | Training | 0.335 | Training × Session | 0.335 | 0.790 | 0.418 | 0.003 | 0.000 | 0.001 | -0.089 |
|  | **Age** | **0.360** | **Training × Age** | **0.368** | **-4.139** | **<.001*** | **0.016** | **-0.011** | **-0.004** | **0.466** |
|  | **Task** | **0.342** | **Training × Task** | **0.345** | **-3.425** | **<.001*** | **0.008** | **-0.005** | **-0.001** | **0.385** |
|  | Age | 0.360 | Session × Age | 0.361 | -0.964 | 0.346 | 0.004 | -0.001 | 0.000 | 0.108 |
|  | Task | 0.342 | Session × Task | 0.339 | 6.542 | <.001* | 0.003 | 0.002 | 0.003 | -0.736 |
|  | **Age** | **0.360** | **Task × Age** | **0.374** | **-10.067** | **<.001*** | **0.012** | **-0.016** | **-0.011** | **1.133** |
|  | Training × Age | 0.368 | Training × Age × Session | 0.367 | 0.802 | 0.451 | 0.006 | -0.001 | 0.002 | -0.090 |
|  | Training × Task | 0.345 | Training × Session × Task | 0.340 | 9.944 | <.001* | 0.005 | 0.004 | 0.007 | -1.119 |
|  | **Task × Age** | **0.374** | **Training × Age × Task** | **0.380** | **-3.205** | **0.002*** | **0.018** | **-0.011** | **-0.002** | **0.361** |
|  | **Task × Age** | **0.374** | **Session × Age × Task** | **0.369** | **7.514** | **<.001*** | **0.006** | **0.003** | **0.006** | **-0.845** |
|  | Training × Age × Task | 0.380 | Training × Session × Age × Task | 0.369 | 11.889 | <.001* | 0.008 | 0.009 | 0.013 | -1.338 |
|  | **Training × Age × Task** | **0.380** | **Individual** | **0.775** | **-19.468** | **<.001*** | **0.180** | **-0.435** | **-0.354** | **2.190** |
|  | **Individual** | **0.775** | **Individual × Task** | **0.886** | **-8.550** | **<.001*** | **0.115** | **-0.137** | **-0.085** | **0.962** |
|  | **Individual** | **0.775** | **Individual × Session** | **0.793** | **-2.531** | **0.016*** | **0.064** | **-0.032** | **-0.004** | **0.285** |
|  |  |  |  |  |  |  |  |  |  |  |
|  |  |  |  |  |  |  |  |  |  |  |
|  |  |  |  |  |  |  |  |  |  |  |
|  |  |  |  |  |  |  |  |  |  |  |
|  |  |  |  |  |  |  |  |  |  |  |
|  |  |  |  |  |  |  |  |  |  |  |
| **Frequency** | **Compared effects and means** | | | | **Statistical results** | | | | | |
|  | **First effect (baseline)** | **Mean** | **Second effect (investigated)** | **Mean** | ***t*-test (*df* = 78)** | ***p* (uncorrected)** | ***SD*** | **95% CI (lower)** | **95% CI (upper)** | ***Cohen's d*** |
| **alpha (8-12 Hz)** | Common | 0.215 | Session | 0.215 | -0.628 | 0.526 | 0.003 | -0.001 | 0.000 | 0.071 |
|  | **Common** | **0.215** | **Task** | **0.219** | **-4.072** | **<.001*** | **0.010** | **-0.007** | **-0.002** | **0.458** |
|  | **Common** | **0.215** | **Age** | **0.264** | **-7.128** | **<.001*** | **0.061** | **-0.062** | **-0.035** | **0.802** |
|  | **Common** | **0.215** | **Training** | **0.223** | **-3.755** | **<.001*** | **0.020** | **-0.013** | **-0.004** | **0.422** |
|  | Training | 0.223 | Training × Session | 0.223 | 0.870 | 0.402 | 0.004 | -0.001 | 0.001 | -0.098 |
|  | **Age** | **0.264** | **Training × Age** | **0.278** | **-4.139** | **<.001*** | **0.030** | **-0.021** | **-0.007** | **0.466** |
|  | Task | 0.223 | Training × Task | 0.226 | -1.874 | 0.051 | 0.011 | -0.005 | 0.000 | 0.211 |
|  | Age | 0.264 | Session × Age | 0.265 | -1.700 | 0.096 | 0.006 | -0.002 | 0.000 | 0.191 |
|  | Task | 0.219 | Session × Task | 0.216 | 7.350 | <.001* | 0.004 | 0.002 | 0.004 | -0.827 |
|  | **Age** | **0.264** | **Task × Age** | **0.268** | **-3.147** | **0.001*** | **0.012** | **-0.007** | **-0.002** | **0.354** |
|  | Training × Age | 0.278 | Training × Age × Session | 0.278 | -0.157 | 0.877 | 0.008 | -0.002 | 0.002 | 0.018 |
|  | Training × Task | 0.226 | Training × Session × Task | 0.218 | 10.767 | <.001* | 0.006 | 0.006 | 0.008 | -1.211 |
|  | Task × Age | 0.278 | Training × Age × Task | 0.278 | 0.084 | 0.943 | 0.013 | -0.003 | 0.003 | -0.009 |
|  | Task × Age | 0.268 | Session × Age × Task | 0.263 | 7.630 | <.001* | 0.006 | 0.004 | 0.007 | -0.858 |
|  | Training × Age × Task | 0.278 | Training × Session × Age × Task | 0.264 | 7.188 | <.001* | 0.017 | 0.010 | 0.017 | -0.809 |
|  | **Training × Age × Task** | **0.278** | **Individual** | **0.810** | **-18.009** | **<.001*** | **0.263** | **-0.591** | **-0.473** | **2.026** |
|  | **Individual** | **0.810** | **Individual × Task** | **0.891** | **-5.391** | **<.001*** | **0.134** | **-0.111** | **-0.051** | **0.607** |
|  | **Individual** | **0.810** | **Individual × Session** | **0.850** | **-4.196** | **<.001*** | **0.085** | **-0.059** | **-0.021** | **0.472** |
|  |  |  |  |  |  |  |  |  |  |  |
|  |  |  |  |  |  |  |  |  |  |  |
|  |  |  |  |  |  |  |  |  |  |  |
|  |  |  |  |  |  |  |  |  |  |  |
|  |  |  |  |  |  |  |  |  |  |  |
|  |  |  |  |  |  |  |  |  |  |  |
| **Frequency** | **Compared effects and means** | | | | **Statistical results** | | | | | |
|  | **First effect (baseline)** | **Mean** | **Second effect (investigated)** | **Mean** | ***t*-test (*df* = 78)** | ***p* (uncorrected)** | ***SD*** | **95% CI (lower)** | **95% CI (upper)** | ***Cohen's d*** |
| **beta (12-30 Hz)** | Common | 0.253 | Session | 0.253 | -1.275 | 0.223 | 0.002 | -0.001 | 0.000 | 0.143 |
|  | **Common** | **0.253** | **Task** | **0.260** | **-9.415** | **<.001*** | **0.006** | **-0.008** | **-0.005** | **1.059** |
|  | **Common** | **0.253** | **Age** | **0.282** | **-7.488** | **<.001*** | **0.035** | **-0.037** | **-0.022** | **0.842** |
|  | **Common** | **0.253** | **Training** | **0.259** | **-2.683** | **0.013*** | **0.019** | **-0.010** | **-0.001** | **0.302** |
|  | Training | 0.259 | Training × Session | 0.259 | -1.114 | 0.272 | 0.004 | -0.001 | 0.000 | 0.125 |
|  | **Age** | **0.282** | **Training × Age** | **0.291** | **-3.639** | **<.001*** | **0.021** | **-0.013** | **-0.004** | **0.409** |
|  | Task | 0.260 | Training × Task | 0.263 | -1.685 | 0.112 | 0.019 | -0.008 | 0.001 | 0.190 |
|  | Age | 0.282 | Session × Age | 0.283 | -1.070 | 0.291 | 0.003 | -0.001 | 0.000 | 0.120 |
|  | Task | 0.260 | Session × Task | 0.257 | 8.152 | <.001* | 0.003 | 0.002 | 0.003 | -0.917 |
|  | **Age** | **0.282** | **Task × Age** | **0.288** | **-6.929** | **<.001*** | **0.008** | **-0.008** | **-0.004** | **0.780** |
|  | Training × Age | 0.291 | Training × Age × Session | 0.292 | -1.157 | 0.257 | 0.006 | -0.002 | 0.001 | 0.130 |
|  | Training × Task | 0.263 | Training × Session × Task | 0.258 | 10.763 | <.001* | 0.004 | 0.004 | 0.006 | -1.211 |
|  | Task × Age | 0.291 | Training × Age × Task | 0.293 | -1.762 | 0.084 | 0.010 | -0.004 | 0.000 | 0.198 |
|  | Task × Age | 0.288 | Session × Age × Task | 0.283 | 10.564 | <.001* | 0.004 | 0.004 | 0.006 | -1.188 |
|  | Training × Age × Task | 0.293 | Training × Session × Age × Task | 0.283 | 12.930 | <.001* | 0.007 | 0.009 | 0.012 | -1.455 |
|  | **Training × Age × Task** | **0.293** | **Individual** | **0.722** | **-16.355** | **<.001*** | **0.233** | **-0.482** | **-0.377** | **1.840** |
|  | Individual | 0.722 | Individual × Task | 0.738 | -1.297 | 0.207 | 0.109 | -0.040 | 0.009 | 0.146 |
|  | **Individual** | **0.722** | **Individual × Session** | **0.802** | **-6.851** | **<.001*** | **0.103** | **-0.103** | **-0.056** | **0.771** |

**Table S2.** Summary of the statistical results of the separate group analysis by frequency band, by age group, and by training group. The *Frequency* column indicates the frequency band. The *Compared effects and means* columns show the two compared effects and their averaged similarity, and indicate which effect was the *baseline* for the comparison and which effect was *investigated* for uniquely contributing to the similarity. The *Statistical results* columns show the results of the statistical comparison. Each row shows one dependent *t*-test comparison. The values of *p* are uncorrected, * shows whether the *p* value remained significant after the FDR (False Discovery Rate) correction, and the **bold text** indicates whether the effect was significantly different from and larger than its baseline.

| **Age** | **Training** | **Frequency** | **Compared effects and means** | | | | **Statistical results** | | | | | |
| --- | --- | --- | --- | --- | --- | --- | --- | --- | --- | --- | --- | --- |
|  |  |  | **First effect (baseline)** | **Mean** | **Second effect (investigated)** | **Mean** | ***t*-test (*df* = 19)** | ***p* (uncorrected)** | ***SD*** | **95% CI (lower)** | **95% CI (upper)** | ***Cohen's d*** |
| **younger** | **control** | **delta**  **(1-4Hz)** | Common | 0.547 | Session | 0.548 | -0.742 | 0.473 | 0.006 | -0.004 | 0.002 | 0.166 |
|  |  |  | **Common** | **0.547** | **Task** | **0.572** | **-6.160** | **<.001*** | **0.018** | **-0.033** | **-0.016** | **1.377** |
|  |  |  | Task | 0.572 | Session × Task | 0.563 | 4.421 | <.001* | 0.009 | 0.005 | 0.013 | -0.989 |
|  |  |  | **Task** | **0.572** | **Individual** | **0.832** | **-8.287** | **<.001*** | **0.140** | **-0.326** | **-0.195** | **1.853** |
|  |  |  | **Individual** | **0.832** | **Individual × Task** | **1.050** | **-4.803** | **<.001*** | **0.203** | **-0.313** | **-0.123** | **1.074** |
|  |  |  | Individual | 0.832 | Individual × Session | 0.816 | 0.781 | 0.478 | 0.092 | -0.027 | 0.059 | -0.175 |
|  |  | **theta**  **(4-7 Hz)** | Common | 0.410 | Session | 0.409 | 1.408 | 0.171 | 0.005 | -0.001 | 0.004 | -0.315 |
|  |  |  | **Common** | **0.410** | **Task** | **0.422** | **-4.903** | **<.001*** | **0.011** | **-0.017** | **-0.007** | **1.096** |
|  |  |  | Task | 0.422 | Session × Task | 0.409 | 7.405 | <.001* | 0.008 | 0.009 | 0.017 | -1.656 |
|  |  |  | **Task** | **0.422** | **Individual** | **0.828** | **-12.407** | **<.001*** | **0.146** | **-0.475** | **-0.338** | **2.774** |
|  |  |  | **Individual** | **0.828** | **Individual × Task** | **0.951** | **-7.276** | **<.001*** | **0.076** | **-0.158** | **-0.088** | **1.627** |
|  |  |  | Individual | 0.828 | Individual × Session | 0.840 | -1.012 | 0.324 | 0.050 | -0.035 | 0.012 | 0.226 |
|  |  | **alpha**  **(8-12 Hz)** | Common | 0.265 | Session | 0.263 | 1.302 | 0.206 | 0.006 | -0.001 | 0.004 | -0.291 |
|  |  |  | Common | 0.265 | Task | 0.264 | 0.386 | 0.703 | 0.010 | -0.004 | 0.006 | -0.086 |
|  |  |  | Task | 0.264 | Session × Task | 0.249 | 8.406 | <.001* | 0.008 | 0.012 | 0.019 | -1.880 |
|  |  |  | **Task** | **0.264** | **Individual** | **0.775** | **-8.633** | **<.001*** | **0.264** | **-0.634** | **-0.387** | **1.930** |
|  |  |  | **Individual** | **0.775** | **Individual × Task** | **0.910** | **-5.353** | **<.001*** | **0.113** | **-0.189** | **-0.083** | **1.197** |
|  |  |  | Individual | 0.775 | Individual × Session | 0.775 | -0.013 | 0.988 | 0.058 | -0.027 | 0.027 | 0.003 |
|  |  | **beta**  **(12-30 Hz)** | Common | 0.318 | Session | 0.318 | -0.451 | 0.645 | 0.005 | -0.003 | 0.002 | 0.101 |
|  |  |  | Common | 0.318 | Task | 0.322 | -1.783 | 0.095 | 0.011 | -0.009 | 0.001 | 0.399 |
|  |  |  | Task | 0.322 | Session × Task | 0.313 | 5.590 | <.001* | 0.007 | 0.006 | 0.013 | -1.250 |
|  |  |  | **Task** | **0.322** | **Individual** | **0.695** | **-5.939** | **<.001*** | **0.281** | **-0.505** | **-0.242** | **1.328** |
|  |  |  | Individual | 0.695 | Individual × Task | 0.732 | -1.367 | 0.196 | 0.120 | -0.093 | 0.020 | 0.306 |
|  |  |  | **Individual** | **0.695** | **Individual × Session** | **0.771** | **-4.039** | **<.001*** | **0.084** | **-0.115** | **-0.036** | **0.903** |
|  |  |  |  |  |  |  |  |  |  |  |  |  |
|  |  |  |  |  |  |  |  |  |  |  |  |  |
|  |  |  |  |  |  |  |  |  |  |  |  |  |
| **Age** | **Training** | **Frequency** | **Compared effects and means** | | | | **Statistical results** | | | | | |
|  |  |  | **First effect (baseline)** | **Mean** | **Second effect (investigated)** | **Mean** | ***t*-test (*df* = 18)** | ***p* (uncorrected)** | ***SD*** | **95% CI (lower)** | **95% CI (upper)** | ***Cohen's d*** |
| **younger** | **training** | **delta**  **(1-4Hz)** | **Common** | **0.384** | **Session** | **0.412** | **-4.357** | **0.002*** | **0.027** | **-0.041** | **-0.014** | **1.000** |
|  |  |  | **Common** | **0.384** | **Task** | **0.403** | **-7.193** | **<.001*** | **0.011** | **-0.024** | **-0.013** | **1.650** |
|  |  |  | **Task** | **0.412** | **Session × Task** | **0.431** | **-4.002** | **0.001*** | **0.022** | **-0.030** | **-0.009** | **0.918** |
|  |  |  | **Task** | **0.431** | **Individual** | **0.629** | **-5.536** | **<.001*** | **0.155** | **-0.272** | **-0.122** | **1.270** |
|  |  |  | Individual | 0.629 | Individual × Task | 0.686 | -1.645 | 0.116 | 0.153 | -0.131 | 0.016 | 0.377 |
|  |  |  | **Individual** | **0.629** | **Individual × Session** | **0.720** | **-3.481** | **0.001*** | **0.114** | **-0.145** | **-0.036** | **0.799** |
|  |  | **theta**  **(4-7 Hz)** | **Common** | **0.439** | **Session** | **0.442** | **-3.298** | **0.002*** | **0.005** | **-0.006** | **-0.001** | **0.757** |
|  |  |  | **Common** | **0.439** | **Task** | **0.465** | **-7.595** | **<.001*** | **0.015** | **-0.034** | **-0.019** | **1.742** |
|  |  |  | Task | 0.465 | Session × Task | 0.459 | 3.400 | 0.003* | 0.008 | 0.002 | 0.010 | -0.780 |
|  |  |  | **Task** | **0.465** | **Individual** | **0.764** | **-7.256** | **<.001*** | **0.179** | **-0.385** | **-0.212** | **1.665** |
|  |  |  | **Individual** | **0.764** | **Individual × Task** | **0.862** | **-3.187** | **0.007*** | **0.134** | **-0.162** | **-0.033** | **0.731** |
|  |  |  | Individual | 0.764 | Individual × Session | 0.787 | -1.484 | 0.164 | 0.069 | -0.057 | 0.010 | 0.340 |
|  |  | **alpha**  **(8-12 Hz)** | **Common** | **0.210** | **Session** | **0.219** | **-3.686** | **0.002*** | **0.011** | **-0.014** | **-0.004** | **0.846** |
|  |  |  | Common | 0.210 | Task | 0.208 | 0.533 | 0.62 | 0.013 | -0.005 | 0.008 | -0.122 |
|  |  |  | Task | 0.219 | Session × Task | 0.204 | 3.641 | 0.003* | 0.017 | 0.006 | 0.023 | -0.835 |
|  |  |  | **Task** | **0.219** | **Individual** | **0.736** | **-7.516** | **<.001*** | **0.300** | **-0.662** | **-0.373** | **1.724** |
|  |  |  | Individual | 0.736 | Individual × Task | 0.775 | -1.138 | 0.261 | 0.148 | -0.110 | 0.033 | 0.261 |
|  |  |  | **Individual** | **0.736** | **Individual × Session** | **0.816** | **-3.154** | **<.001*** | **0.110** | **-0.133** | **-0.027** | **0.724** |
|  |  | **beta**  **(12-30 Hz)** | Common | 0.290 | Session | 0.291 | -1.381 | 0.178 | 0.006 | -0.004 | 0.001 | 0.317 |
|  |  |  | Common | 0.290 | Task | 0.289 | 0.606 | 0.544 | 0.008 | -0.003 | 0.005 | -0.139 |
|  |  |  | Task | 0.291 | Session × Task | 0.279 | 4.608 | <.001* | 0.012 | 0.007 | 0.018 | -1.057 |
|  |  |  | **Task** | **0.291** | **Individual** | **0.728** | **-9.794** | **<.001*** | **0.194** | **-0.530** | **-0.343** | **2.247** |
|  |  |  | Individual | 0.728 | Individual × Task | 0.717 | 0.410 | 0.672 | 0.114 | -0.044 | 0.066 | -0.094 |
|  |  |  | **Individual** | **0.728** | **Individual × Session** | **0.813** | **-3.037** | **<.001*** | **0.123** | **-0.145** | **-0.026** | **0.697** |
|  |  |  |  |  |  |  |  |  |  |  |  |  |
|  |  |  |  |  |  |  |  |  |  |  |  |  |
|  |  |  |  |  |  |  |  |  |  |  |  |  |
|  |  |  |  |  |  |  |  |  |  |  |  |  |
|  |  |  |  |  |  |  |  |  |  |  |  |  |
| **Age** | **Training** | **Frequency** | **Compared effects and means** | | | | **Statistical results** | | | | | |
|  |  |  | **First effect (baseline)** | **Mean** | **Second effect (investigated)** | **Mean** | ***t*-test (*df* = 19)** | ***p* (uncorrected)** | ***SD*** | **95% CI (lower)** | **95% CI (upper)** | ***Cohen's d*** |
| **older** | **control** | **delta**  **(1-4Hz)** | Common | 0.338 | Session | 0.334 | 4.497 | 0.001* | 0.004 | 0.002 | 0.005 | -1.006 |
|  |  |  | **Common** | **0.338** | **Task** | **0.366** | **-6.358** | **<.001*** | **0.020** | **-0.037** | **-0.019** | **1.422** |
|  |  |  | Task | 0.366 | Session × Task | 0.348 | 11.852 | <.001* | 0.006 | 0.014 | 0.020 | -2.650 |
|  |  |  | **Task** | **0.366** | **Individual** | **0.693** | **-7.621** | **<.001*** | **0.192** | **-0.417** | **-0.237** | **1.704** |
|  |  |  | **Individual** | **0.693** | **Individual × Task** | **0.984** | **-7.167** | **<.001*** | **0.182** | **-0.377** | **-0.206** | **1.603** |
|  |  |  | Individual | 0.693 | Individual × Session | 0.664 | 1.949 | 0.062 | 0.066 | -0.002 | 0.059 | -0.436 |
|  |  | **theta**  **(4-7 Hz)** | Common | 0.331 | Session | 0.331 | 0.462 | 0.647 | 0.007 | -0.002 | 0.004 | -0.103 |
|  |  |  | **Common** | **0.331** | **Task** | **0.339** | **-3.163** | **<.001*** | **0.011** | **-0.013** | **-0.003** | **0.707** |
|  |  |  | Task | 0.339 | Session × Task | 0.326 | 6.954 | <.001* | 0.009 | 0.009 | 0.018 | -1.555 |
|  |  |  | **Task** | **0.339** | **Individual** | **0.784** | **-10.473** | **<.001*** | **0.190** | **-0.534** | **-0.356** | **2.342** |
|  |  |  | **Individual** | **0.784** | **Individual × Task** | **0.933** | **-4.740** | **<.001*** | **0.140** | **-0.214** | **-0.083** | **1.060** |
|  |  |  | Individual | 0.784 | Individual × Session | 0.792 | -0.481 | 0.634 | 0.072 | -0.041 | 0.026 | 0.107 |
|  |  | **alpha**  **(8-12 Hz)** | Common | 0.348 | Session | 0.344 | 5.493 | <.001* | 0.003 | 0.003 | 0.006 | -1.228 |
|  |  |  | Common | 0.348 | Task | 0.351 | -0.799 | 0.445 | 0.019 | -0.012 | 0.006 | 0.179 |
|  |  |  | Task | 0.351 | Session × Task | 0.334 | 9.807 | <.001* | 0.008 | 0.013 | 0.021 | -2.193 |
|  |  |  | **Task** | **0.351** | **Individual** | **0.876** | **-7.476** | **<.001*** | **0.314** | **-0.672** | **-0.378** | **1.672** |
|  |  |  | **Individual** | **0.876** | **Individual × Task** | **1.001** | **-3.775** | **0.001*** | **0.148** | **-0.194** | **-0.056** | **0.844** |
|  |  |  | Individual | 0.876 | Individual × Session | 0.877 | -0.059 | 0.956 | 0.068 | -0.033 | 0.031 | 0.013 |
|  |  | **beta**  **(12-30 Hz)** | Common | 0.313 | Session | 0.312 | 0.408 | 0.718 | 0.005 | -0.002 | 0.003 | -0.091 |
|  |  |  | Common | 0.313 | Task | 0.314 | -0.770 | 0.452 | 0.010 | -0.006 | 0.003 | 0.172 |
|  |  |  | Task | 0.314 | Session × Task | 0.303 | 8.568 | <.001* | 0.006 | 0.009 | 0.014 | -1.916 |
|  |  |  | **Task** | **0.314** | **Individual** | **0.728** | **-8.875** | **<.001*** | **0.209** | **-0.512** | **-0.316** | **1.985** |
|  |  |  | Individual | 0.728 | Individual × Task | 0.732 | -0.127 | 0.897 | 0.114 | -0.057 | 0.050 | 0.028 |
|  |  |  | **Individual** | **0.728** | **Individual × Session** | **0.815** | **-3.125** | **0.002*** | **0.124** | **-0.145** | **-0.029** | **0.699** |
|  |  |  |  |  |  |  |  |  |  |  |  |  |
|  |  |  |  |  |  |  |  |  |  |  |  |  |
|  |  |  |  |  |  |  |  |  |  |  |  |  |
|  |  |  |  |  |  |  |  |  |  |  |  |  |
|  |  |  |  |  |  |  |  |  |  |  |  |  |
|  |  |  |  |  |  |  |  |  |  |  |  |  |
|  |  |  |  |  |  |  |  |  |  |  |  |  |
| **Age** | **Training** | **Frequency** | **Compared effects and means** | | | | **Statistical results** | | | | | |
|  |  |  | **First effect (baseline)** | **Mean** | **Second effect (investigated)** | **Mean** | ***t*-test (*df* = 19)** | ***p* (uncorrected)** | ***SD*** | **95% CI (lower)** | **95% CI (upper)** | ***Cohen's d*** |
| **older** | **training** | **delta (1-4Hz)** | Common | 0.313 | Session | 0.313 | -0.426 | 0.682 | 0.007 | -0.004 | 0.003 | 0.095 |
|  |  |  | **Common** | **0.313** | **Task** | **0.326** | **-4.502** | **<.001*** | **0.013** | **-0.020** | **-0.007** | **1.007** |
|  |  |  | Task | 0.326 | Session × Task | 0.320 | 2.202 | 0.033* | 0.013 | 0.000 | 0.012 | -0.492 |
|  |  |  | **Task** | **0.326** | **Individual** | **0.623** | **-6.862** | **<.001*** | **0.193** | **-0.387** | **-0.206** | **1.534** |
|  |  |  | **Individual** | **0.623** | **Individual × Task** | **0.783** | **-5.304** | **<.001*** | **0.135** | **-0.224** | **-0.097** | **1.186** |
|  |  |  | Individual | 0.623 | Individual × Session | 0.621 | 0.123 | 0.915 | 0.060 | -0.026 | 0.030 | -0.028 |
|  |  | **theta (4-7 Hz)** | Common | 0.294 | Session | 0.291 | 5.072 | <.001* | 0.003 | 0.002 | 0.004 | -1.134 |
|  |  |  | **Common** | **0.294** | **Task** | **0.298** | **-2.217** | **0.037*** | **0.009** | **-0.008** | **0.000** | **0.496** |
|  |  |  | Task | 0.298 | Session × Task | 0.286 | 7.028 | <.001* | 0.008 | 0.009 | 0.016 | -1.572 |
|  |  |  | **Task** | **0.298** | **Individual** | **0.722** | **-10.558** | **<.001*** | **0.180** | **-0.508** | **-0.340** | **2.361** |
|  |  |  | **Individual** | **0.722** | **Individual × Task** | **0.796** | **-3.474** | **0.002*** | **0.095** | **-0.119** | **-0.029** | **0.777** |
|  |  |  | **Individual** | **0.722** | **Individual × Session** | **0.753** | **-2.105** | **0.05*** | **0.064** | **-0.061** | **0.000** | **0.471** |
|  |  | **alpha (8-12 Hz)** | Common | 0.285 | Session | 0.282 | 2.280 | 0.038 | 0.005 | 0.000 | 0.005 | -0.510 |
|  |  |  | Common | 0.285 | Task | 0.283 | 0.705 | 0.483 | 0.010 | -0.003 | 0.006 | -0.158 |
|  |  |  | Task | 0.283 | Session × Task | 0.267 | 9.743 | <.001* | 0.007 | 0.013 | 0.020 | -2.179 |
|  |  |  | **Task** | **0.283** | **Individual** | **0.849** | **-13.379** | **<.001*** | **0.189** | **-0.655** | **-0.478** | **2.992** |
|  |  |  | Individual | 0.849 | Individual × Task | 0.873 | -1.205 | 0.246 | 0.088 | -0.065 | 0.018 | 0.269 |
|  |  |  | **Individual** | **0.849** | **Individual × Session** | **0.932** | **-5.999** | **<.001*** | **0.061** | **-0.111** | **-0.054** | **1.341** |
|  |  | **beta (12-30 Hz)** | Common | 0.244 | Session | 0.245 | -0.814 | 0.421 | 0.007 | -0.005 | 0.002 | 0.182 |
|  |  |  | Common | 0.244 | Task | 0.246 | -1.217 | 0.254 | 0.009 | -0.007 | 0.002 | 0.272 |
|  |  |  | Task | 0.246 | Session × Task | 0.235 | 5.937 | <.001* | 0.009 | 0.007 | 0.015 | -1.328 |
|  |  |  | **Task** | **0.246** | **Individual** | **0.738** | **-9.346** | **<.001*** | **0.235** | **-0.601** | **-0.381** | **2.090** |
|  |  |  | Individual | 0.738 | Individual × Task | 0.771 | -1.728 | 0.106 | 0.085 | -0.073 | 0.007 | 0.386 |
|  |  |  | **Individual** | **0.738** | **Individual × Session** | **0.808** | **-3.827** | **0.001*** | **0.082** | **-0.109** | **-0.032** | **0.856** |

**Sensitivity power analysis**

**Table S3.** Minimal effect sizes to reach 80%, 90%, or 95% power for alpha = .05 with the present sample sizes for the present hypothesis test.

| **Age group** | **Training group** | ***N*** | **Power = 80%** | **Power = 90%** | **Power = 95%** |
| --- | --- | --- | --- | --- | --- |
|  |  |  | **alpha = .05** | **alpha = .05** | **alpha = .05** |
| All | All | 79 | 0.319 | 0.369 | 0.411 |
| Younger | Control | 20 | 0.66 | 0.765 | 0.85 |
|  | Training | 19 | 0.68 | 0.787 | 0.875 |
| Older | Control | 20 | 0.66 | 0.765 | 0.85 |
|  | Training | 20 | 0.66 | 0.765 | 0.85 |

**Figure S1**. Sensitivity power analysis curve showing the reached statistical power as a function of effect size. The sample sizes are 79 (top), 20 (middle), 19 (bottom), and alpha = .05.
